# Transit time flow management as a management strategy in high-risk groups undergoing coronary artery bypass grafting

**DOI:** 10.1186/s13019-025-03408-8

**Published:** 2025-03-21

**Authors:** John Kucera, John Duggan, Alex Peters, Gregory Trachiotis

**Affiliations:** 1 Department of Surgery, Walter Reed National Military Medical Center, Bethesda, MD USA; 2 Division of Cardiothoracic Surgery and Heart Center, Washington DC Veterans Affairs Medical Center, Washington, DC USA

**Keywords:** Transit time flow management, Coronary artery bypass grafting, Diabetes, ESRD, On Pump Coronary Artery Bypass Graft, Off Pump Coronary Artery Bypass Graft

## Abstract

**Background:**

We evaluated the surgical outcomes in three groups of individuals with diabetes mellitus (DM), end-stage renal disease (ESRD), and on (ONCAB) vs. off-pump (OPCAB) coronary artery bypass grafting (CABG). We also examined the changes in intraoperative decision-making when ultrasound and transit-time flow measurement was utilized in the operating room. This study will aim to identify the utility of HFUS and TTFM in high-risk patient categories.

**Methods:**

Data from the multicenter REQUEST (Registry for Quality assessment with ultrasound imaging and TTFM measurement in cardiac bypass surgery) had recently been compiled in three separate papers examining outcomes in patients with DM, ESRD, and on vs. off-pump bypass grafting. Data was extrapolated to determine the impact of HFUS and TTFM in patients with diabetes, ESRD, ONCAB and OPCAB. The primary outcome measured in in the REQUEST study is any change in planned surgical procedure. Secondary end points include rate of changes, coronary targets, completed grafts, and in-hospital morbidity and mortality.

**Results:**

Outcomes were predicated upon patient population surveyed. The REQUEST registry reported 1016 individuals who underwent CABG. For individuals with DM, any surgical change to the coronary target was slightly lower, measured at a change rate of 11.6% vs. 9.5% (OR 0.80, 95% CI 0.53–1.21, *P* = 0.288). In diabetics, the aortic component of the operation underwent a higher rate of surgical strategy change with TTFM compared to without (10.2% vs. 6.4%, OR 1.67, 95% CI 1.06–2.65; *P* = 0.026). In patients with ESRD, TTFM increased the rate of strategy changes compared to no TTFM (33.7% vs. 24.3%, 95% CI 1.01–2.48, *P* = 0.047) and number of revisions per graft (7.0% vs. 3.4%, OR 2.14, 95% CI 1.17–3.71). In the 1016 individuals who underwent CABG, 402 (39.6%) underwent OPCAB and 614 (60.4%) undergoing ONCAB. When TTFM and HFUS were utilized, OPCAB resulted in greater number of strategy changes for aortic portion of the procedure (14.7% vs. 3.4%, OR 4.03, CI 2.32–7.20) without a difference in coronary target or graft revision. In the REQUEST study, in-hospital mortality was published at 0.6%.

**Conclusions:**

TTFM use demonstrates a statistically significant impact on intra-operative decision making and operative strategy changes in patients with concomitant ESRD, DM and who are undergoing OPCAB relative to ONCAB. This difference in OPCAB vs. ONCAB may be related to higher mean graft flows in OPCAB in the setting of a standardized TTFM cutoff for determination of graft quality. This data cumulatively suggests there a role for TTFM in CABG, namely due to its positive impact on outcome and statistically significant impact on intra-operative decision making.

## Background

Coronary artery bypass grafting was introduced approximately 5 decades ago and has resulted in cardiac surgeons pursuing a reduction of perioperative and postoperative adverse events. This pursuit has resulted in evolution in long-term outcomes with the introduction of internal mammary arterial grafting [1], technical advancement, and constantly improving medical management aimed at secondary prevention of progressive coronary disease. The cumulative impact of this multidisciplinary approach to coronary disease and its ultimate surgical management has resulted in a progressive improvement in long-term outcomes such as mortality and graft patency.

Various techniques employed intraoperatively continue to be standard-of-care for CABG. This includes adequate graft selection, identification of aorta free of atherosclerosis, and less invasive techniques. The REQUEST study is a multicenter, prospective, international registry evaluating the impact of Transit-time flow management to improve coronary graft assessment and successfully identify areas of disease-free aorta using ultrasound [2]. It enrolled 1046 individuals in 7 centers across North America, with primary end point being the frequency of change in the previously planned surgical procedure [2]. Secondary end points consisted of the rationale and rate of operative change, major adverse cardiac and cerebrovascular events (MACCE) [2], new hemodialysis requirement and re-operation for bleeding [2]. Surgeons were trained to use and interpret these intraoperative studies, with MAP set at 80mHg during assessment and the HFUS probe frequency standardized at 15 MHz [2]. Assessment of TTFM findings were further standardized, however the operating surgeon was allowed to determine if a graft should be revised based on findings obtained during TTFM [2].

The results of the REQUEST study were subsequently published in 2020 and noted that any surgical change was made in 25.2% of patients who underwent TTFM based on perceived abnormal TTFM and HFUS findings [2]. It is important to note that these findings were found in the setting of a detailed visual inspection where there had been no appreciable suspicion of a diseased aorta, or abnormalities in either in-situ graft conduit or final complete graft. In 12.5% of patients, in contrast, a change to the surgical plan was implemented based on grossly abnormal visual or palpable findings in either the aorta or completed graft [2]. Changes related to the aorta were implemented in a total of 9.9% of patients (80 of 806) and 74% of these changes were predicated on abnormal HFUS findings [2].

This data was subsequently examined via subgroup analysis in the setting of end stage renal disease [3], diabetes mellitus [4], and on pump coronary artery bypass graft (ONCAB) vs. off pump coronary artery bypass graft (OPCAB) [5, 6]. However, there has not been a descriptive analysis of all three manuscripts taken in concert with the original REQUEST data to elucidate the physiologic correlates in each population in concert with providing an easily accessible compilation of available data as it relates to the role of TTFM and HUS as a valuable tool in optimizing outcomes in coronary artery bypass grafting.

## Methods

A total of seven manuscripts were curated from the literature, including sub-analyses of the REQUEST study describing the impact of TTFM and HFUS on individuals with DM, ESRD and ONCAB vs. OPCAB. Baseline patient characteristics in each study were noted, and results were stratified based on the presence of each specific pathology. Relevant characteristics of individuals were subsequently assimilated and a compilation of results from each sub-analysis tabulated. There was no additional statistical analysis performed in this summary and review of each paper, as their respective results had been previously validated.

### Surgeon characteristics and TTFM assessment

To participate in the study, cardiac surgeons were required to have prior training verified externally with at least 20 CABG procedures involving TTFM and HFUS [2]. They were trained in the interpretation of TTFM and HFUS, and documented their prior plan for cannulation site, bypass grafting technique, cross clamp site, and number of anastomoses [2]. Following the procedure, the ultimate operative technique was recorded, and the results compared. During assessment with TTFM and HFUS, MAP was standardized at 80mmHg [2]. Changes prompting evaluation of completed grafts for potential revision included (1) decrease in diastolic filling (< 70% for left-sided and 50% for right-sided coronary vessels), (2) pulsatility index > 5, (3) arterial grafts with < 15 mL/min and venous grafts with < 20mL/min of blood flow [2]. It is important to note that ultimately the decision to implement a change in the operative technique was left at the discretion of the operating surgeon and there was no standardized protocol-driven basis for this process.

### REQUEST cohort and definition of primary outcome

Ultimately, 1046 individuals were enrolled at 7 participating centers across Europe and North America. Their individual characteristics, including comorbid conditions, were collected. Change in surgical strategy was defined as any deviation from the previously established plan. While these changes could be related to findings based on above characteristics from TTFM and HFUS, they could also be due to palpable findings. Aortic changes were further stratified based on if they occurred during the aortic cannulation site, aortic cross-clamp location, or proximal anastomosis location. Coronary target changes were also noted, as were revision of completed grafts either in a primary (technical issue with the anastomosis) or secondary (graft kinking or inadequate graft length), need for additional grafts, or revision of the conduit itself. These results were then statistically analyzed and any *P*-value of < 0.05 considered to be significant.

#### Diabetes mellitus in REQUEST cohort

Following the release of the original REQUEST study, Duggan et al. released a sub-analysis of the REQUEST results to determine if individuals with DM underwent a higher number or rate of surgical strategy changes in comparison to individuals without DM [4]. Their analysis astutely considered that individuals with DM are a well-known unique population undergoing CABG and experience a higher rate of death from cardiac causes and elevated incidence of nonfatal myocardial infarction (MI) [7]. There is such a stark association with DM and CAD that cardiovascular risk is noted to be the same in individuals who had a prior MI and do not have DM [8]. They have a higher incidence of DM-related comorbid conditions such as greater atherosclerotic burden, microvascular disease, chronic kidney disease, and long-term outcomes after CABG are inferior to individuals without DM [9]. Their post-operative non-cardiac outcomes are also inferior relative to the general population, with a greater risk of cerebrovascular event, sternal wound infection, and all-cause mortality [8]. However, CABG remains the preferred revascularization technique given inferior outcome when percutaneous coronary intervention (PCI) is considered [9, 10].

The REQUEST study contained 402 individuals with DM, with 128 of these being insulin dependent (Table 1). These individuals were extrapolated from the original data, and demographic information including age, BMI, prior MI, revascularization history, and other comorbid conditions were tabulated [2]. Changes in surgical strategy was defined in a similar fashion to the REQUEST study, as were conduit changes and anastomosis revisions. This was compared to individuals without DM and used to calculate an odds-ratio (OR) [5]. The rate of bilateral internal mammary artery (BIMA) was also recorded, as was use of the radial artery. Adverse events which occurred both in-hospital for individuals with and without diabetes were also tabulated.

**Table 1:** Demographic and clinical characteristics of patients with and without diabetes enrolled in the REQUEST study

Pre-operative Patient Variables	Non-Diabetic (*n* = 614)	Diabetic (*n* = 402)	*P*
Age (years)	66.1 ± 9.8	65.6 ± 9.1	0.358
Sex (female)	67 (10.9)	76 (18.9)	< 0.001**
[Sex (male)]	547 (89.1)	326 (81.1)	-
BMI (kg/m2) ^a^	27.3 (24.9, 30.4)	28.7 (25.8, 32.5)	< 0.001**
Prior Myocardial Infarction	193 (31.4)	138 (34.3)	0.336
History of coronary revascularization	127 (20.7)	105 (26.1)	0.044*
Prior CABG	2 (0.3)	5 (1.2)	0.120
Prior PCI	125 (20.4)	104 (25.9)	0.040*
Stroke history	34 (5.5)	28 (7.0)	0.353
Hypertension	389 (63.4)	335 (83.3)	< 0.001**
Hyperlipidemia	301 (49.0)	257 (63.9)	< 0.001**
COPD	38 (6.2)	39 (9.7)	0.039*
History of carotid/peripheral vascular intervention	28 (4.6)	17 (4.2)	0.802
CKD/ESRD	45 (7.3)	50 (12.4)	0.006*
Atrial fibrillation	20 (3.3)	13 (3.2)	0.984
LVEF < 30% ^b^	6/587 (1.0)	18/391 (4.6)	< 0.001**
Missing	27	11	-
CCS angina classification III-IV ^b^	239/582 (41.1)	164/390 (42.1)	0.760
Missing	32	12	-
NYHA classification III-IV ^b^	109/558 (19.5)	81/377 (21.5)	0.467
Missing	56	25	-
Left main involvement ^b^	273/491 (55.6)	164/306 (53.6)	0.580
Missing	123	96	-

BMI, body mass index; CABG, coronary artery bypass grafting; COPD, chronic obstructive pulmonary disease; CKD/ESRD; Chronic Kidney Disease/End Stage Renal Disease; LVEF, left ventricular ejection fraction; CCS, Canadian Cardiovascular Society grading of angina pectoris; NYHA, New York Heart Association functional classification of heart failure

Data are presented as number and percentage, mean ± standard deviation, or median (interquartile range)

*p < 0.05

**p < 0.01

^a^ BMI was unknown for 1 patient

^b^ Patients with missing data for a given variable were not considered when calculating percentages or performing group comparisons for these specific variables

#### End stage renal disease

A sub analysis of the REQUEST study was initiated like that undertaken for DM in 2021. This queried the outcomes of CABG in patients with chronic kidney disease (CKD) in addition to end stage renal disease (ESRD) and compared them to those enrolled in the REQUEST study with normal renal function, in retrospective fashion [3]. Like the REQUEST study, the primary end point was intraoperative surgical strategy change with secondary end point being post-protamine TTFM parameters [3]. This resulted in the collation of 95 patients with CKD and ESRD in contrast to 921 with normal renal function [3]. Kriskal-Wallis tests were implemented to determine relationships between CKD and ESRD diagnosis and TTFP parameters, which were further differentiated based on graft type and target arterial territory. Only single conduit to single coronary targets were included in this analysis, and grafts with less than 75% of TTFM parameters tabulated or available were also excluded [4]. In addition, data regarding diastolic filling percentage was also absent in more than 25% of grafts in the ESRD population, so this was excluded from analysis [3].

### Off versus on-pump coronary artery bypass grafting

Like the sub-analyses conducted above in individuals with DM and ESRD undergoing TTFM and HFUS during CABG, a sub-analysis evaluating the impact and rate of operative strategy change in individuals undergoing on vs. off-pump CABG [5, 6]. The REQUEST study was utilized for this sub-analysis, and strategy changes were defined in similar fashion to the above. Patients undergoing OPCAB vs. ONCAB were compared based on preoperative characteristics, operative variables, frequency of use of HFUS or TTFM at each site of interrogation [6]. Quantitative and qualitative data was collected to determine the number of strategy changes which transpired because of HFUS or TTFM, and rates of major in-hospital cardiac, vascular, or cerebrovascular events were also noted. Revision rates were compared based on the location of the distal anastomosis in either the inferior, anterior or lateral cardiac arterial distribution [6].

Following the collection of this data, categorical data was presented as a proportion while continuous data was reported as either a mean or median for parametric vs. non-parametric variables, respectively. Incidence of surgical changes, clinical outcomes, demographic data was presented as an odds ratio with 95% confidence intervals [6].

## Results

### Diabetes mellitus

Any surgical change to the coronary target was slightly lower in diabetic patients, 11.6% vs. 9.5% (OR 0.80, 95% CI 0.53–1.21, *P* = 0.288). It is notable that HFUS resulted in a change in 9.8% of diabetic patients, compared to only 1.5% based simply on visual or tactile feedback. Individuals with diabetes were noted to have a higher rate of change in surgical strategy related to the aorta after evaluation with HFUS (10.2% vs. 6.4%, OR 1.67, 95% CI 1.06–2.65; *P* = 0.026) (Table 2).^4^ This resulted most in a change to the site of the proximal anastomosis, but alterations in location of cannulation and cross-clamp site were also appreciated. Notably, visual and tactile feedback resulted in a lower rate of surgical strategy changes relative to non-diabetic patients (4.0% vs. 4.6%; OR 0.53, 95% CI 0.26–1.11) [4]. In situ conduit strategy changes were noted in 2.2% of individuals relative to 1.5% in those without diabetes (OR 1.54, 95% CI 0.61–3.91), *P* = 0.361).^4^

**Table 2:** Rates of surgical changes from preoperative plan in patients with and without diabetes

Surgical Changes	Non-Diabetic (*n* = 614)	Diabetic (*n* = 402)	Odds Ratio (95% CI)	*P*
Any strategy change	151/614 (24.6%)	105/402 (26.1%)	1.08 (0.81–1.45)	0.584
HFUS/TTFM	109/614 (17.8%)	88/402 (21.9%)	1.30 (0.95–1.78)	0.103
Visual/tactile	28/614 (4.6%)	16/402 (4.0%)	0.87 (0.46–1.63)	0.657
Unclassified	28/614 (4.6%)	10/402 (2.5%)	0.53 (0.26–1.11)	0.089
Changes related to the aorta				
Any surgical change (all patients)	39/614 (6.4%)	41/402 (10.2%)	1.67 (1.06–2.65)	0.026*
HFUS	34/614 (5.5%)	40/402 (10.0%)	-	-
Visual/tactile	3/614 (0.5%)	1/402 (0.2%)	-	-
Unclassified	2/614 (0.3%)	0/402 (0.0%)	-	-
Changes related to in situ conduits				
Any surgical change (all patients)	9/614 (1.5%)	9/402 (2.2%)	1.54 (0.61–3.91)	0.361
HFUS	5/614 (0.8%)	5/402 (1.2%)	-	-
Visual/tactile	3/614 (0.5%)	3/402 (0.7%)	-	-
Unclassified	1/614 (0.2%)	1/402 (0.2%)	-	-
Changes related to coronary targets				
Any surgical change (all patients)	71/614 (11.6%)	38/402 (9.5%)	0.80 (0.53–1.21)	0.288
HFUS	42/614 (6.8%)	31/402 (7.7%)	-	-
Visual/tactile	9/614 (1.5%)	2/402 (0.5%)	-	-
Unclassified	21/614 (3.4%)	6/402 (1.5%)	-	-
Changes related to completed grafts				
Any surgical change (all patients)	50/614 (8.1%)	29/402 (7.2%)	0.88 (0.55–1.41)	0.589
HFUS/TTFM	33/614 (5.3%)	18/402 (4.5%)	-	-
Visual/tactile	16/614 (2.6%)	11/402 (2.7%)	-	-
Unclassified	5/614 (0.8%)	1/402 (0.2%)	-	-

Note: One individual patient can have one or more surgical changes

Data are presented as number of patients with surgical change / number of patients undergoing intraoperative assessment (%)

*p < 0.05

**p < 0.01

Completed graft alterations occurred at a higher rate in diabetic patients, 8.1% vs. 7.2% (OR 0.88; 95% CI 0.55–1.41, *P* = 0.589).^4^ Again, it was found that HFUS and TTFM correlated with a higher rate of change at 5.3%, compared to decisions based on visual or tactile feedback which was found to be 2.6%. Amongst diabetic patients it was also appreciated that more surgical changes occurred in those undergoing on-pump procedures relative of off-pump, however this was not a difference that was found in those without diabetes (2.1% vs. 0.4%, OR 0.21, 95% CI 0.03–1.70, *P* = 0.107). Again, it was demonstrated that HFUS and TTFM resulted in a greater rate of change in contrast to visual or tactile feedback in both on and off-pump CABG in those with diabetes [4].

HFUS and TTFM also had a differential impact based on whether individuals with diabetes underwent OPCAB vs. ONCAB.(Tables 3 and 4) Approximately 226 patients underwent ONCAB relative to 176 who underwent OPCAB. Any strategy change occurred at a higher rate in OPCAB in those with diabetes, with 23% in ONCAB relative to 30.1% in OPCAB (OR 1.44, 95% CI 0.92–2.25, *P* = 0.108).^4^ HFUS and TTFM resulted in a higher rate of change in both OPCAB and ONCAB in those with diabetes relative to visual feedback. ONCAB in those with diabetes who underwent HFUS and TTFM resulted in 17.7% rate of strategy change in contrast to 5.3% with visual feedback alone. OPCAB had similar findings, with HFUS and TTFM resulting in 27.3% of individuals undergoing any strategy change in contrast to only 2.3% with visual feedback alone. Similar findings based on HFUS and TTFM relative to visual exam were appreciated in ONCAB vs. OPCAB regarding aortic changes, conduit, coronary targets and completed grafts.

**Table 3:** Rates of surgical changes from preoperative plan in patients with diabetes: off versus on pump

Surgical Changes	Diabetic			
	On-pump (*n* = 226)	Off-pump (*n* = 176)	Odds Ratio (95% CI)	*P*
Any strategy change	52/226 (23.0%)	53/176 (30.1%)	1.44 (0.92–2.25)	0.108
HFUS/TTFM	40/226 (17.7%)	48/176 (27.3%)	1.74 (1.08–2.81)	0.021*
Visual/tactile	12/226 (5.3%)	4/176 (2.3%)	0.42 (0.13–1.31)	0.112
Unclassified	6/226 (2.7%)	4/176 (2.3%)	0.85 (0.24–3.07)	0.807
Changes related to the aorta				
Any surgical change (all patients)	10/226 (4.4%)	31/176 (17.6%)	4.62 (2.20–9.71)	< 0.001**
HFUS	9/226 (4.0%)	31/176 (17.6%)	-	-
Visual/tactile	1/226 (0.4%)	0/176 (0.0%)	-	-
Unclassified	0/226 (0.0%)	0/176 (0.0%)	-	-
Changes related to in situ conduits				
Any surgical change (all patients)	9/226 (4.0%)	0/176 (0.0%)	NE	0.007*
HFUS	5/226 (2.2%)	0/176 (0.0%)	-	-
Visual/tactile	3/226 (1.3%)	0/176 (0.0%)	-	-
Unclassified	1/226 (0.4%)	0/176 (0.0%)	-	-
Changes related to coronary targets				
Any surgical change (all patients)	26/226 (11.5%)	12/176 (6.8%)	0.56 (0.28–1.15)	0.111
HFUS	22/226 (9.7%)	9/176 (5.1%)	-	-
Visual/tactile	2/226 (0.9%)	0/176 (0.0%)	-	-
Unclassified	3/226 (1.3%)	3/176 (1.7%)	-	-
Changes related to completed grafts				
Any surgical change (all patients)	16/226 (7.1%)	13/176 (7.4%)	1.05 (0.49–2.24)	0.906
HFUS/TTFM	9/226 (4.0%)	9/176 (5.1%)	-	-
Visual/tactile	7/226 (3.1%)	4/176 (2.3%)	-	-
Unclassified	1/226 (0.4%)	0/176 (0.0%)	-	-

Note: One individual patient can have one or more surgical changes

Data are presented as number of patients with surgical change / number of patients (%)

*p < 0.05

**p < 0.01

**Table 4:** Rates of surgical changes from preoperative plan in patients without diabetes: off versus on pump

Surgical Changes	Without Diabetes			
	On-pump (*n* = 388)	Off-pump (*n* = 226)	Odds Ratio (95% CI)	*P*
Any strategy change	76/388 (19.6%)	75/226 (33.2%)	2.04 (1.40–2.96)	< 0.001**
HFUS/TTFM	52/388 (13.4%)	57/226 (25.2%)	2.18 (1.43–3.31)	< 0.001**
Visual/tactile	22/388 (5.7%)	6/226 (2.7%)	0.45 (0.18–1.14)	0.084
Unclassified	13/388 (3.4%)	15/226 (6.6%)	2.05 (0.96–4.39)	0.060
Changes related to the aorta				
Any surgical change (all patients)	11/388 (2.8%)	28/226 (12.4%)	4.85 (2.36–9.94)	< 0.001**
HFUS	7/388 (1.8%)	27/226 (11.9%)	-	-
Visual/tactile	3/388 (0.8%)	0/226 (0.0%)	-	-
Unclassified	1/388 (0.2%)	1/226 (0.4%)	-	-
Changes related to in situ conduits				
Any surgical change (all patients)	8/388 (2.1%)	1/226 (0.4%)	0.21 (0.03–1.70)	0.107
HFUS	5/388 (1.3%)	0/226 (0.0%)	-	-
Visual/tactile	2/388 (0.5%)	1/226 (0.4%)	-	-
Unclassified	1/388 (0.3%)	0/226 (0.0%)	-	-
Changes related to coronary targets				
Any surgical change (all patients)	41/388 (10.6%)	30/226 (13.3%)	1.30 (0.78–2.14)	0.312
HFUS	26/388 (6.7%)	16/226 (7.1%)	-	-
Visual/tactile	7/388 (1.8%)	2/226 (0.9%)	-	-
Unclassified	8/388 (2.1%)	13/226 (5.8%)	-	-
Changes related to completed grafts				
Any surgical change (all patients)	28/388 (7.2%)	22/226 (9.7%)	1.39 (0.77–2.49)	0.271
HFUS/TTFM	16/388 (4.1%)	17/226 (7.5%)	-	-
Visual/tactile	12/388 (3.1%)	4/226 (1.8%)	-	-
Unclassified	3/388 (0.8%)	2/226 (0.9%)	-	-

Note: One individual patient can have one or more surgical changes

Data are presented as number of patients with surgical change / number of patients (%)

*p < 0.05

**p < 0.01

#### ESRD

ESRD and CKD was identified in approximately 95 individuals in the REQUEST database. Comorbid conditions such as stroke (11.6% vs. 5.5%, *P* = 0.019), diabetes, (53% vs. 38%, *P* = 0.006), COPD (16% vs. 7%, *P* = 0.002), and reduced left ventricular ejection fraction (8.7% vs. 1.8%, *P* = 0.001) were more common with ESRD [3]. The severity of either Canadian Cardiovascular Society angina or NYHA functional classification did not differ between these groups [3]. Unfortunately, certain comorbid conditions and female sex did not have adequate sample size to achieve statistical significance. These are tabulated in Table 5.

**Table 5:** Demographic information of patients with ESRD and CKD in the REQUEST study

Demographic and clinical characteristics	Normal renal function (*N* = 921)	CKD/ESRD (*N* = 95)	*P*-value
Age (years)	65.7 ± 9.4	67.5 ± 10.3	0.064
Sex (female)	130 (14.1)	13 (13.7)	0.91
Body mass index (kg/m2)a	27.7 (25.2–31.0)	28.1 (25.6–31.9)	0.37
Prior myocardial infarction	293 (31.8)	38 (40.0)	0.11
History of coronary revascularization	203 (22.0)	29 (30.5)	0.061
Prior coronary artery bypass grafting	6 (0.7)	1 (1.1)	0.50
Prior percutaneous coronary intervention	200 (21.7)	29 (30.5)	0.050
History of stroke	51 (5.5)	11 (11.6)	0.019
Hypertension	644 (69.9)	80 (84.2)	0.003
Hyperlipidemia	508 (55.2)	50 (52.6)	0.64
Diabetes mellitus	352 (38.2)	50 (52.6)	0.006
Chronic obstructive pulmonary disease	62 (6.7)	15 (15.8)	0.002
Carotid artery stenosis	82 (8.9)	11 (11.6)	0.39
Peripheral vascular disease	82 (8.9)	16 (16.8)	0.013
History of carotid/peripheral vascular intervention	40 (4.3)	5 (5.3)	0.60
Atrial fibrillation	28 (3.0)	5 (5.3)	0.23
Left-ventricular ejection fraction < 30%b	16 (1.8)	8 (8.7)	0.001
Missing	35	3	
Canadian Cardiovascular Society angina classification			0.096
0	102 (11.6)	18 (19.4)	
I–II	410 (46.6)	39 (41.9)	
III–IV	367 (41.8)	36 (38.7)	
Missing	42	2	
New York Heart Association functional classificationb			0.20
I	324 (38.3)	25 (28.4)	
II	356 (42.0)	40 (45.5)	
III	141 (16.7)	18 (20.5)	
IV	26 (3.1)	5 (5.7)	
Left main involvement	400 (54.7)	37 (56.1)	0.83

Adapted from Rosenfield *et al*

HFUS and TTFM resulted in a higher rate of any strategy change in CKD and ESRD patients, including when compared to visual inspection alone (26.3% vs. 10.5%) (Table 6). This trend continued when examining changes relative to the aorta in contrast to visual inspection (10.5% vs. 1.1%), and coronary targets (7.4% vs. 1.1%). Changes related to in-situ conduits happened at a lower rate with TTFM and HFUS compared to visual inspection alone (1.1% vs. 3.2%) and was equivocal when examining changes related to grafts in each patient [3].

**Table 6:** Strategy changes in patients with ESRD or CKD in the REQUEST study

	Normal renal function (*N* = 921)	CKD/ESRD (*N* = 95)	Odds ratio (95% CI)	*P*-value	
Any strategy change	224/921 (24.3)	32/95 (33.7)	1.58 (1.01–2.48)	0.047	
HFUS/TTFM	172/921 (18.7)	25/95 (26.3)	1.56 (0.96–2.53)	0.075	
Visual/tactile feedback	34/921 (3.7)	10/95 (10.5)	3.08 (1.47–6.43)	0.003	
Unclassified change	35/921 (3.8)	3/95 (3.2)	0.83 (0.25–2.74)	0.75	
Changes related to the aorta					
Any surgical change	69/921 (7.5)	11/95 (11.6)	1.62 (0.74–3.23)	0.16	
HFUS	64/921 (6.9)	10/95 (10.5)			
Visual/tactile feedback	3/921 (0.3)	1/95 (1.1)			
Unclassified change	2/921 (0.2)	0/95 (0.0)			
Changes related to in situ conduits					
Any surgical change	14/921 (1.5)	4/95 (4.2)	2.84 (0.67–9.30)	0.079	
HFUS and/or TTFM	9/921 (1.0)	1/95 (1.1)			
Visual/tactile feedback	3/921 (0.3)	3/95 (3.2)			
Unclassified change	2/921 (0.2)	0/95 (0.0)			
Changes related to coronary targets					
Any surgical change	98/921 (10.6)	11/95 (11.6)	1.10 (0.51–2.16)	0.73	
HFUS	66/921 (7.2)	7/95 (7.4)			
Visual/tactile feedback	10/921 (1.1)	1/95 (1.1)			
Unclassified change	24/921 (2.6)	3/95 (3.2)			
Changes related to grafts (per patient)					
Any surgical change	68/921 (7.4)	11/95 (11.6)	1.64 (0.75–3.28)	0.16	
HFUS and/or TTFM	44/921 (4.8)	7/95 (7.4)			
Visual/tactile feedback	20/921 (2.2)	7/95 (7.4)			
Unclassified change	6/921 (0.7)	0/95 (0.0)			
Changes related to grafts (per graft)					
Any surgical change	83/2433 (3.4)	17/242 (7.0)	2.14 (1.17–3.71)	0.008	
HFUS and/or TTFM	53/2433 (2.2)	9/242 (3.7)			
Visual/tactile feedback	23/2433 (0.9)	8/242 (3.3)			
Unclassified change	7/2433 (0.3)	0/242 (0.0)			

Adapted from Rosenfield *et al*

Patients with ESRD were found to undergo OPCAB at a higher rate than those who had normal renal function (48.4% vs. 38.7%, *P* = 0.064)^3^, and less frequent use of bilateral internal mammary arterial grafts (33.7% vs. 44.2%, *P* = 0.049). Regarding changes in operative strategy, there were significantly more changes overall in ESRD patients than those without (33.7% vs. 24.3%, OR 1.58, 95% CI 1.01–2.48, *P* = 0.047). ESRD portended a greater number of graft revision (7.0% vs. 3.4%, OR 2.14, 95% CI 0.96–2.53, *P* = 0.075). HFUS and TTFM was found to demonstrate an improvement in parameters in those with ESRD in nearly 89% of patients, in contrast to 82% in those without ESRD or CKD [3].

### OPCAB vs. ONCAB

Within the REQUEST cohort there were 402 patients (39.6%) who underwent OPCAB and 614 (60.4%) who underwent ONCAB [6]. All these individuals underwent median sternotomy and had similar operative times. However, OPCAB had higher LIMA use (98.3% vs. 95.8%) and demonstrated higher use of the radial artery (37.8% vs. 12.7%).^6^ There was lower use of bilateral internal mammary artery in OPCAB, at 25.1% vs. 34.0%. There were overall more arterial conduits employed in OPCAB (62.7% vs. 55.4%) and greater use of HFUS in OPCAB (88.3% vs. 73.5%).^6^ HFUS was used less commonly to interrogate coronary targets (33.6% vs. 54.7%) and completed grafts (46.0% vs. 67.9%) in OPCAB vs. ONCAB, respectively [6].

In the absence of HFUS, there was no difference in changes to coronary targets, however with the addition of HFUS there were more in OPCAB (28.6%) than in ONCAB (19.9%)^6^. HFUS appeared to enhance the ability of the operative surgeon to identify inadequate targets, and subsequently identify completed grafts intra-oepratively. The increase in changes to coronary targets in OPCAB were primarily due to the identification of coronary calcification on HFUS, with 45.2% of initial targets in OPCAB and 47.8% in ONCAB noted to have calcification at target location [6]. Following completion, there was no appreciable difference in revision rates of completed grafts between OPCAB and ONCAB on a per-patient or per-graft basis.

Surgical strategy changes occurred more frequently to the ascending aorta more frequently in OPCAB vs. ONCAB (14.7% vs. 3.4%, OR 4.03, 95% CI 2.32–7.20). This was attributable to changes demonstrated on HFUS, which led to 98.3% of alterations in OPCAB and 76.2% in ONCAB [6]. This was substantially higher than demonstrated based on visual or tactile feedback, with only 19% of patients undergoing ONCAB and 0% of OPCAB having a change in surgical plan. Overall, aortic changes were most related to finding an alternate proximal anastomosis site in OPCAB (81.4%) compared to only 33.3% in ONCAB [6].

Strategy changes for in situ conduits was less common in OPCAB vs. ONCAB (0.2% vs. 2.8%, OR 0.09, 95% CI 0.01–0.56) [6]. Of these, conduit changes in ONCAB were attributable to findings demonstrated on HFUS (58.8%) whereas only 23.5% were due to either a technical error or visual appreciation of diminutive caliber of conduit. In this instance, the literature is somewhat misleading in noting that visual or tactile feedback accounted for 100% of conduit changes in OPCAB, but only one patient who underwent OPCAB had any change to conduit. Adverse events were also similar, with no difference in overall cerebrovascular or cardiovascular events. It is important to note that Leviner et al. found that mean graft flow is higher in ONCAB vs. OPCAB, (32 vs. 28 mL/min, respectively, for all grafts [*P* < 0.001]; 30 vs. 27 mL/min for arterial grafts [*P* = 0.002]; and 35 vs. 31 mL/min for venous grafts [*P* = 0.006], respectively) [5]. This is importantly in the absence of a differential TTFM cutoff in OPCAB vs. ONCAB.

## Discussion

Collation of data obtained from the REQUEST study demonstrated that in individuals with diabetes, surgeons were more likely to alter their strategy for the aortic portion based on HFUS results [5]. In addition, diabetic patients did not receive bilateral internal mammary artery grafts in comparison to non-diabetics, and there was an increase in the total number of grafts [11]. This is likely in an effort to mitigate the known association of BIMA grafts and sternal wound infection or poor healing. This population typically also have a greater incidence of early-onset coronary disease, and more technically complex lesions. Studies have noted an increase in atherosclerotic coronary burden, more extensive disease, and a resultant challenge in distal coronary target accuracy in the absence of HFUS or TTFM [12]. Henceforth, HFUS and TTFM play a valuable role in indentifying adequate targets in this population with an increased risk of complex coronary disease burden. Overall, diabetics have worse outcomes after CABG although trials such as the Bypass Angioplasty Revascularization Investigation and Synergy have demonstrated the superiority of CABG in this population relative to PCI [13]. Consequentially, CABG is recommended over PCI by multiple institutions such as the American Heart Association and American College of Cardiology Foundation [14].

Although CABG is the preferred method of revascularization for individuals with diabetes, it presents several unique constraints in this population. Foremost amongst these is the result of diabetics having inferior outcomes when compared to the non-diabetic population following CABG [15]. They also have more complex coronary disease, and an increased risk of stroke resulting from a greater atheroma burden in the ascending aorta [16]. The application of HFUS and TTFM when assessing the ideal site of aortic cannulation, cross-clamping, and adequacy of coronary grafts with appropriate proximal and distal anastomoses has clear utility in this population [17]. Multiple prior studies have demonstrated the superior ability of HFUS to identify aortic atheroma relative to palpation and visual inspection alone. This contributes to diabetics having a greater rate of surgical strategy changes regarding cannulation, cross-clamp location and anastomosis location in the REQUEST study [5]. However, this did not culminate in a decrease in stroke risk in this population and given the lack of long-term outpatient follow up data it is difficult to definitively state that HFUS has a benefit in this situation. The data does, however, support that HFUS and TTFM did have a role in improving location choice in coronary anastomosis and assessing the technical quality of the completed anastomosis. Diabetics are less likely to have bilateral internal mammary arterial grafts given the risk of sternal wound infection, although use in the REQUEST study was higher than seen in the general population (25% vs. 6.7%)^5^. Saphenous vein grafts remain the most common in this population, which was also true in the REQUEST study.

Those with ESRD and CKD also have an elevated perioperative risk profile when compared to individuals with normal renal function. Although there is clear evidence arguing for the superiority of CABG in this population (Roberts et al.) there remains a reluctance for surgeons to perform CABG in these individuals [18]. Unlike DM, there is a dearth of quality literature describing changes in outcomes and postoperative course in this population, emphasizing the importance of the retrospective analysis of REQUEST data [3]. This review demonstrated that these individuals also had a 39% greater rate of surgical strategy change when compared to those with normal renal function [3]. Coronary lesions have long been assumed to be more complex and diffuse in those with ESRD and CKD [19], associated with both direct uremic damage and comorbid hypertension and diabetes mellitus, culminating in difficulty with accurate surgical planning [19]. There was a greater chance of a more technically difficult anastomosis, or an unanticipated coronary lesion, oftentimes only identified once HFUS and TTFM was employed intraoperatively [3]. Furthermore, although these ESRD patients had a greater rate of surgical strategy change based on visual or tactile perception (7.4% vs. 2.2% in normal renal function), confirmatory testing with HFUS and TTFM was able to confirm suspicion of need for graft revision [3].

Independent of those who underwent a revision of their coronary graft based on visual or tactile perception, lesions identified only with HFUS and TTFM were observed at a 41% greater rate in patients with ESRD than in those with normal renal function [3]. Although this was not a statistically significant finding in the retrospective analysis due to small sample size, it does demonstrate that further studies with a greater sample size may demonstrate that this difference does indeed exist. Again, there was a statistically significant alteration in grafts based on visual and tactile feedback which was confirmed in each instance with HFUS and TTFM. HFUS and TTFM may therefore be able to identify anatomic constraints that would otherwise be difficult to perceive, both in the coronaries and ascending aorta.

Nearly 11% of ESRD individuals underwent an aortic surgical strategy change based on HFUS interrogation, with no patients having a stroke [3]. ESRD portends a greater risk of arterial calcification and subsequent stroke risk during cross-clamping and cannulation of the aorta [20], with HFUS mitigating this somewhat and ensuring safe handling of the aorta which should also be further investigated [21]. Following completion of coronary grafts, flow rates and pulsatility were like those with normal renal function based on TTFM. This may be attributable to the utility of HFUS and TTFM in identifying appropriate coronary anastomosis sites, as it was seen across multiple conduit types and coronary targets.

Evaluation of HFUS and TTFM in individuals undergoing OPCAB vs. ONCAB demonstrated a differing pattern of strategy changes and graft alterations predicated on OPCAB vs. ONCAB status. Individuals who underwent OPCAB had a rate of surgical strategy change related to the aortic portion of the procedure are 4 times that of ONCAB [6]. Conduit alterations occurred twice as frequently in arterial conduit in contrast to venous grafts [6].

In OPCAB patients, the sub-analysis demonstrated 14.7% of individuals underwent a strategy change to the ascending aorta, with nearly 82% of these changes being an alteration in the location of the proximal anastomosis [5]. This contrasted with 3.4% of those undergoing ONCAB having a similar change to the aortic portion of the procedure [6]. This was dictated by HFUS results in 98.3% of these strategy changes, with none of the causative atheroma identified by either visual or tactile inspection [5]. Examining the causality behind the difference in OPCAB vs. ONCAB patients yields that HFUS was employed in 88.3% of those undergoing OPCAB in contrast to 73.5% in ONCAB [6]. Furthermore, individuals with DM were more prevalent in OPCAB (44% vs. 37%) and patients who had atherosclerotic disease in the ascending aorta underwent OPCAB given a perceived increase in stroke risk [5] (Tables 3 and 4).

These results demonstrate that HFUS has a clear benefit in stroke prevention in both OPCAB and ONCAB, with atheroma detected using this imaging modality that were not appreciated on visual or tactile feedback alone [6]. This is supported by a 0.5% stroke rate when HFUS was employed in contrast to a recent analysis demonstrating a 1.1% stroke rate [6]. The sub-analysis also noted that arterial conduits underwent more revisions than venous. Although evidence supports long-term patency in arterial grafts being superior to venous, there is a technical learning curve with more difficult anastomotic construction. Arterial grafts also tend to spasm, which will alter the ability of TTFM to be accurately interpreted, with HFUS being a useful tool to confirm TTFM measurements which may indicate a need for revision of arterial grafts.

Inferior cardiac grafts were also more likely to undergo revision, at a rate 1.8 times higher than anterior or lateral territories [6]. This is likely due to the technical difficulties associated with this distribution, notably with the RCA. Even amongst expert cardiac surgeons, the REQUEST study sub-analysis showed that in OPCAB 5.1% of inferior grafts required revision, with 81.0% of these revisions showing improvement on repeat TTFM and HFUS [5].

Notably within this sub-analysis, there was no direct comparison made between OPCAB and ONCAB, nor were any comparisons made about outcomes with and without HFUS and TTFM. This review does not seek to compare the efficacy of HFUS and TTFM between these groups, rather discuss them independently within a single manuscript. There was no follow-up documented beyond the hospital course, and decision to make surgical changes was reliant upon the outcomes of expert cardiac surgeons who had rates of BIMA and complete revascularization which was greater than that documented in the community at large.

### Limitations

Long-term data was limited in each sub-analysis because of limitations of the original REQUEST data. This makes it challenging to determine the true benefit of HFUS and TTFM, especially when taken in the context of certain groups not possessing adequate sample size to achieve statistical significance. Furthermore, this study was not designed as a propensity-matched cohort, limiting the ability to compare HFUS and TTFM against cases where it was not used. Furthermore, long-term data regarding outcomes was not available. It was not a randomized controlled trial and is therefore difficult to definitively state that there is a clear benefit to TTFM and HFUS as a result. The surgeons who participated in the REQUEST study were experienced and considered to be well-trained in the use of HFUS and TTFM, which may limit the generalizability of this data. In addition, there is a chance of bias due to surgeon-dependent changes based on HFUS and TTFM results. Each sub-analysis was also extrapolated from a study which was not originally designed to evaluate individuals with DM, ESRD and OPCAB vs. ONCAB. This limits the amount of patient-specific data which is available, such as hemoglobin A1C. There is also no data on long-term outcomes associated with surgical strategy change rates beyond in-hospital patient course.

## Conclusion

This review sought to collate the results of three sub-analyses of the REQUEST study, a prospective protocol seeking to demonstrate the utility of HFUS and TTFM use in CABG. In individuals with diabetes mellitus, it was found that HFUS may have an impact in selecting aortic cannulation, cross-clamp and proximal anastomosis site. The use of HFUS and TTFM demonstrated a greater rate of change to aortic portions of the procedure, coronary targets and completed grafts when compared to visual and tactile feedback alone. Similar findings were noted in individuals with ESRD and CKD, which is likely due to the more complex coronary disease found in these two populations. TTFM and HFUS also had utility in ONCAB vs. OPCAB, notably resulting in a three-times greater rate of aortic strategy changes, accurately selecting coronary targets, and confirming that both graft revision was necessary and sufficient. This study was not designed to compare the efficacy or utility of HFUS and TTFM between the above sub-groups. It does, however, suggest that based on rates of change and ability to assay the quality of completed grafts intra-operatively that there may be a benefit to HFUS and TTFM in high-risk patient groups. Additional studies are needed to compare the efficacy of HFUS and TTFM to visual or tactile feedback in graft quality, and long-term data is needed to note benefit to patients. The summation of this data from each sub-analysis suggests that HFUS and TTFM may have a beneficial role in CABG, notably in high-risk populations.

## Data Availability

No datasets were generated or analysed during the current study.
